# Supplementary material for: Evidence supported by Mendelian randomization: impact on inflammatory factors in knee osteoarthritis
Source: Front Med (Lausanne). 2024 May 28;11:1382836. doi: 10.3389/fmed.2024.1382836 (PMC11165061; doi:10.3389/fmed.2024.1382836)
Supplement: Supplementary file 1 [file Data_Sheet_1.ZIP › data/GCST90274775_5e6/775.leaveoneout.pdf]

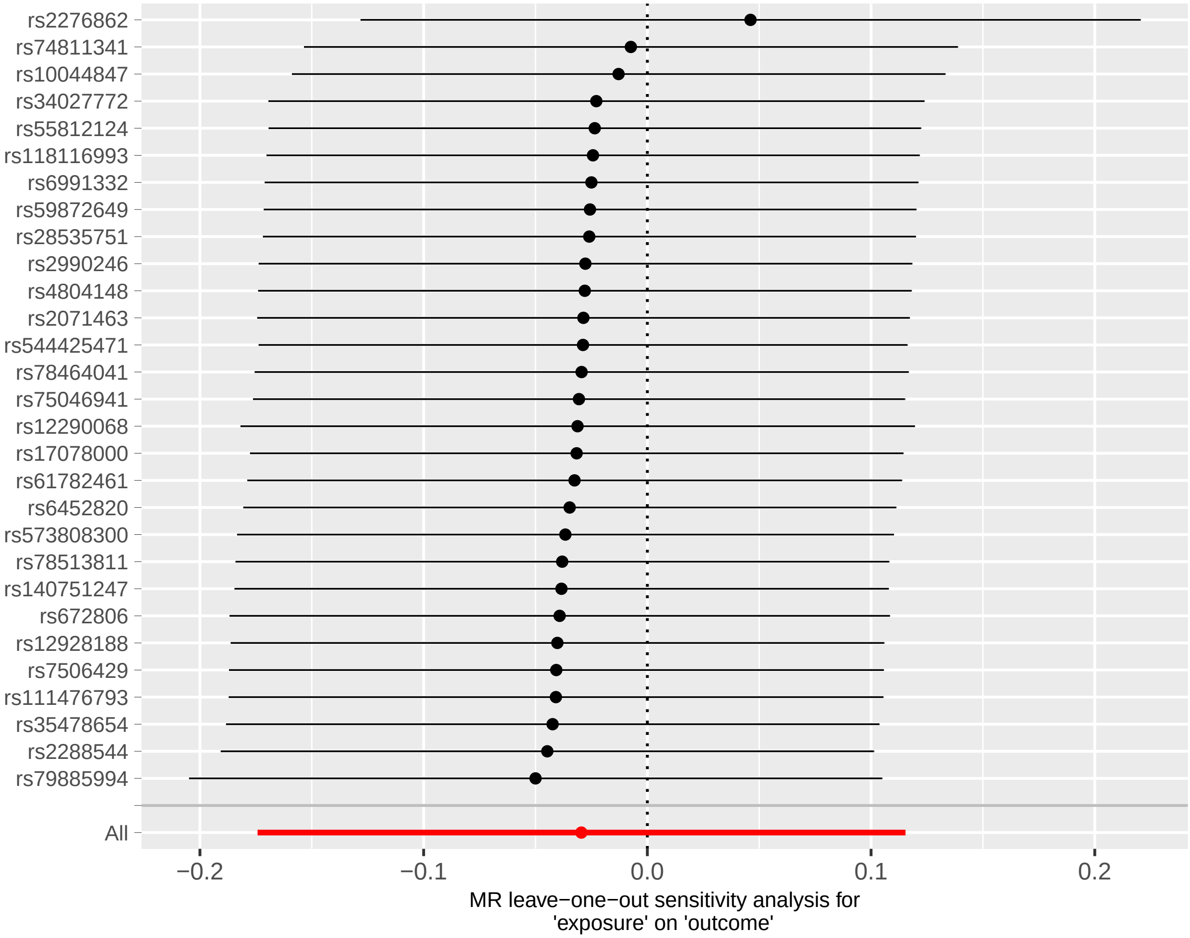
rs2276862
rs74811341
rs10044847
rs34027772
rs55812124
rs118116993
rs6991332
rs59872649
rs28535751
rs2990246
rs4804148
rs2071463
rs544425471
rs78464041
rs75046941
rs12290068
rs17078000
rs61782461
rs6452820
rs573808300
rs78513811
rs140751247
rs672806
rs12928188
rs7506429
rs111476793
rs35478654
rs2288544
rs79885994
All
−0.2
−0.1
0.0
0.1
0.2
MR leave−one−out sensitivity analysis for
'exposure' on 'outcome'
